# Inverse Association between Serum 25-hydroxyvitamin D Levels and Risk of Suspected Non-Alcoholic Fatty Liver Disease in Obese Population

**DOI:** 10.3390/ijerph18168682

**Published:** 2021-08-17

**Authors:** Eunjung Park, Eun Young Park

**Affiliations:** 1 Department of Public Health Sciences, Graduate Scholl of Hanyang University, 222, Wangsimni-ro, Seongdong-gu, Seoul 04763, Korea; eunjungsays@gmail.com; 2 Division of Cancer Prevention, National Cancer Control Institute, National Cancer Center, 323, Ilsan-ro, Ilsandong-gu, Goyang-si 10408, Korea

**Keywords:** vitamin D, serum 25-hydroxyvitamin D, obesity, liver injury, non-alcoholic fatty liver disease (NAFLD)

## Abstract

Background: Worldwide, vitamin D deficiency is a public health issue and the prevalence of obesity and non-alcoholic fatty liver disease (NAFLD) are rapidly increasing. There are a limited number of studies assessing the association between serum levels of 25-hydroxyvitamin D (25(OH)D) and NAFLD risk in obese population. Objective: We evaluated the associations between serum 25(OH)D levels and risk of suspected NAFLD after stratification by obesity using data from the Korea National Health and Nutrition Examination Survey (KNHANES) 2008–2014. Methods: This study included 25,755 subjects without significant alcohol consumption for the serum alanine aminotransferase (ALT) and hepatic steatosis index (HSI) analyses (8922 subjects for the serum gamma-glutamyl transferase (GGT) and fatty liver index (FLI) analyses), based on a cross-sectional study design. Serum 25(OH)D levels were measured using a Gamma counter with radioimmunoassay. A survey logistic regression model was applied to estimate ORs and 95% CIs. Restricted cubic smoothing splines were applied to evaluate nonlinear associations. Results: The risk of suspected NAFLD was reduced per unit of natural log-transformed serum 25(OH)D concentration in obese individuals (OR [95% (CI)]; for ALT, 0.80 [0.67, 0.96]; for GGT, 0.70 [0.49, 0.99; for FLI, 0.68 [0.47, 1.01]; for HSI, 0.70 [0.56, 0.87]). The ORs [95% CI] of suspected NAFLD changed across the quartiles: for serum ALT, from 1.02 [0.85, 1.23] to 0.72 [0.59, 0.87]; for serum GGT, from 0.79 [0.56, 1.13] to 0.64 [0.44, 0.92]; for FLI, from 0.98 [0.67, 1.44] to 0.70 [0.48, 1.02]; and for HSI, from 0.91 [0.73, 1.14] to 0.65 [0.52, 0.81] with dose–response relationships (all *p* for trend < 0.01). Conclusions: This study suggests that vitamin D sufficiency for public health should be emphasized in order to prevent adverse health effects in obese populations.

## 1. Introduction

Worldwide, vitamin D deficiency is one of the most important public health issues and its prevalence in general populations is approximately 36% in the United States, 61% in Canada, 92% in Northern Europe, 45–98% in Asia, 31% in Australia and 56% in New Zealand [1]. Vitamin D is a lipophilic vitamin which is involved in calcium homeostasis, bone metabolism and immune function. Vitamin D can be obtained mainly by synthesis from exposure to ultraviolet B (UVB) radiation on skin, since natural dietary sources of vitamin D are limited for most individuals [2,3]. Vitamin D is metabolized into 25-hydroxyvitamin D (25(OH)D) in the liver which is its major circulating metabolite in human body [3]. Several epidemiologic studies show that low serum levels of 25(OH)D are associated with adverse health outcomes, such as metabolic syndrome [4], diabetes [5,6,7], chronic liver disease [8,9], cancer [1,7,10], cardiovascular disease [7,10,11] and all-cause mortality [11].

Non-alcoholic fatty liver disease (NAFLD) is the most common liver disease, and its burden has rapidly increased [12]. The prevalence of NAFLD in general populations over the age of 19 is approximately 24.13% in North America, 23.71% in Europe and 25.37% in Asia [13]. The increase in the prevalence of NAFLD has been paralleling the increase of obesity prevalence worldwide [14]. Obesity is known to be independently related to NAFLD. A large cohort study of Korea showed that the hazard ratios (95% confidence intervals) for NAFLD in overweight (body mass index (BMI): 23.0–24.9 kg/m^2^) and obese (BMI: ≥25.0 kg/m^2^) participants were 2.15 (2.06–2.26) and 3.55 (3.37–3.74), compared with normal weight (BMI: 18.5–22.9 kg/m^2^) participants [15].

NAFLD is diagnosed when there is imaging or histological evidence of hepatic fat accumulation in individuals without significant alcohol consumption [16,17]. There is growing evidence that NAFLD is associated with type 2 diabetes mellitus [18,19,20], dyslipidemia, hypertension, cardiovascular disease [21] and cancers [22]. Recently, several studies have suggested that low serum 25(OH)D levels are positively associated with risk of NAFLD diagnosed by ultrasonography or by biopsy [23,24], and might be an independent predictor of the severity of NAFLD. On the other hand, clinical trials on the effectiveness of oral vitamin D supplementation on NAFLD showed inconclusive results [25,26]. Therefore, the associations between serum 25(OH)D levels and NAFLD risk are still uncertain.

Vitamin D deficiency is more common in Korea. According to the 2014 Korean National Health and Nutrition Examination Survey (KNHANES), the means of serum 25(OH)D for the surveyed population were 17.28 ng/mL in males and 15.68 ng/mL in females. Since 2008, when the measurement of serum vitamin D began, vitamin D deficiency has been continuously intensifying [27]. To the best of our knowledge, there are few studies assessing the association between vitamin D deficiency and NAFLD risk in obese population.

Here, we hypothesized that vitamin D sufficiency might reduce NAFLD risk in an obese population. To test this hypothesis, we conducted a cross-sectional study to investigate the association between serum 25(OH)D levels and risk of unexplained elevation of serum liver enzymes and NAFLD indices (i.e., hepatic steatosis index (HSI), fatty liver index (FLI)) in obese population after stratification by obesity, in KNHANES, nationally representative data.

## 2. Methods

### 2.1. Data Source and Study Population

We used data from the KNHANES 2008–2014, conducted annually using a rolling sampling design which is a complex, stratified, multistage, and probability clustering for a representative sample of the general population of Republic of Korea. The subjects were informed during the surveys that they had been randomly selected as a household and that they would take part in the nationally representative survey conducted by the Korean Ministry of Health and Welfare. 

The serum 25(OH)D test was performed in all subjects aged 10 years or older from 2008 to 2012, but we randomly extracted 1500 subjects from 2013 to 2014 by survey district, sex, and age (10 s, 20 s, 30 s, 40 s, 50 s, 60 s or older), out of the whole survey population.

Participants provided questionnaires for information on demographic characteristics, which included sex, age, cigarette smoking, alcohol consumption, family income level, education level, and physical activity. The health examination was performed by trained medical staff for all participants, and it was composed of a physical examination and biochemical measurements that follow a standardized procedure. Participants provided written informed consent on KNHANES. 

The KNHANES was approved by the institutional review board of the Korea Centers for Disease Control and Prevention (KCDC). 

We reported according to the Strengthening the Reporting of Observational Studies in Epidemiology (STROBE) guidelines for the reporting of this cross-sectional study.

KNHANES subjects aged more than 19 years from 2008 to 2014 were included in this study, with data on serum 25(OH)D and liver enzymes (AST, ALT and GGT). Among 46,759 subjects aged 19 years or older, 18,636 subjects were eligible for inclusion, after we excluded 12,137 subjects without serum 25(OH)D levels, and 6499 drinkers (i.e., alcohol consumption status: >21 standard drinks/week for males, >14 standard drinks/week for females). We also excluded subjects who had liver diseases (i.e., positive tests for anti-hepatitis B virus (HBV) or anti-hepatitis C virus (HCV) antibodies, or self-reported histories of HBV, HCV, liver cirrhosis, or liver cancer), and subjects with missing data for covariates (e.g., smoking status, alcohol consumption status, family income, education level, physical activity, and BMI). The final study subjects were 25,755 for serum ALT and hepatic steatosis index (HSI), and 8922 for serum GGT and fatty liver index (FLI) (serum GGT was measured only in 2010~2011). 

**Figure 1:**
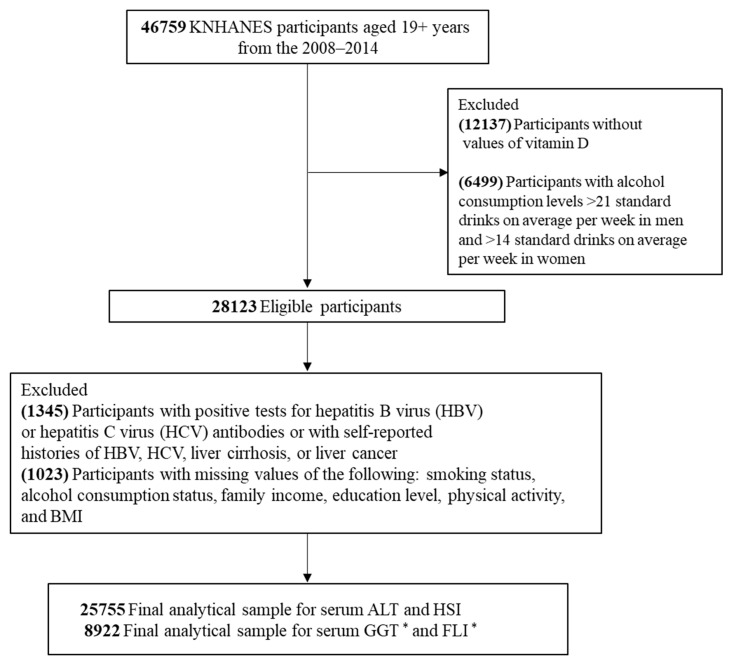
Flow diagram showing study sample derivation. KNHANES: Korea National Health and Nutrition Examination Survey, BMI: body mass index, ALT: alanine aminotransferase, HSI: hepatic steatosis index, GGT: serum gamma-glutamyl transferase, FLI: fatty liver index. * Information on data for GGT and FLI is presented separately, hence serum GGT was measured only in 2010~2011.

A flow diagram that shows how we derived the study sample is presented in Figure 1.

### 2.2. Suspected NAFLD

We defined elevation of serum ALT (ALT > 30 U/L for males and >19 U/L for females) and GGT (GGT >51 U/L for males and >33 U/L for females) [28] and two NAFLD indices (i.e., FLI > 60 and HSI > 36) [21,29] as the ‘suspected NAFLD’. The FLI and HSI were yielded as the following equation: FLI = (0.953× loge [triacylglycerol (mmol/L) ×88.5]) + (0.139 × BMI [kg/m^2^]) + (0.718 × loge GGT [IU/L]) + (0.053 × waist circumference [cm]) − 15.745; HSI = 8 × ALT (IU/L) /AST (IU/L) + BMI (kg/m^2^) + 2 (if type 2 diabetes) + 2 (if female sex) [21,29]. 

### 2.3. Assessment of Serum 25(OH)D and Serum Liver Enzymes

To measure the serum 25(OH)D levels and liver enzymes, blood samples were taken with standard commercial evacuated tubes containing sodium heparin (Vacutainers (BD, Franklin Lakes, NJ, USA)). Serum 25(OH)D levels were assessed using a Gamma counter (1470 WIZARD, PerkinElmer, Finland) with radioimmunoassay (DiaSorin, Stillwater, MN, USA) and was within the standard deviation (SD) from 2008 to 2014 (KCDC, 2009, 2010, 2011, 2012, 2013, 2014, 2015). The NeoDin Medical Institute achieved the Korea Occupational Safety and Health Administration (Korea OSHA) program and also completed the German External Quality Assessment Scheme and the U.S. CDC program.

Serum AST, ALT and GGT were measured using an autobiochemical analyzer (Hitachi 7600, Hitachi High-Technologies Corporation, Ibaraki, Japan). The reagents were pureauto S AST (2010–2012, Daiichi Pure Chemicals, Tokyo, Japan; 2013, 2016, Sekisui Chemical CO. Ltd., Osaka, Japan), pureauto S ALT (2010–2012, Daiichi Pure Chemicals, Tokyo, Japan; 2013, 2016, Sekisui Chemical CO. Ltd., Osaka, Japan), and pureauto S GGT (Sekisui Chemical CO. Ltd., Osaka, Japan).

### 2.4. Statistical Analyses 

Due to skewed distribution of serum 25(OH)D levels, they were natural log-transformed and categorized into quartiles according to the distribution of serum 25(OH)D among the final analytical samples for serum ALT and HSI (i.e., samples from 2008 to 2014). We conducted the Rao-Scotti chi-square test to assess differences in the potential confounders between cases and controls as follows: sex, age, smoking status, alcohol consumption, physical activity, family income level, education level, BMI [28,30], and survey year. We also performed Student’s *t*-test to examine the differences in serum 25(OH)D levels between two groups. 

Smoking status was classified into never smokers (those who has not smoked in their lifetime), former smokers (those who smoked in the past but do not smoke now), and current smokers (those who are currently smoking). We categorized alcohol consumption status as nondrinkers or moderate drinkers (≤21 standard drinks/week for males and ≤14 standard drinks/week for females). We assessed physical activity by the average frequencies (days per month) of medium- or high-strength physical activity, and physical activity was classified as ≥5 days/week or <5 days/week. The categorization for education level was as follows: elementary school or less, middle school, high school, or college or more. Based on the household income of the sample households presented in the KNHANES report from the Korea Centers for Disease Control and Prevention, household income was classified into quartiles. We grouped obesity categories as normal weight (BMI < 23 kg/m^2^), overweight (BMI = 23–24.9 kg/m^2^), and obesity (BMI ≥ 25 kg/m^2^), according to the World Health Organization standards for Asians [31].

Associations between serum 25(OH)D levels and suspected NAFLD were investigated to estimate ORs and 95% CIs, applying survey logistic regression models. The serum 25(OH)D levels were categorized into quartiles; the ORs for each elevated serum liver enzyme and the NAFLD indices were estimated as follows. Model 1 was adjusted for age (continuous variable) and sex (only in the overall dataset) and model 2 was further adjusted for level of family income, education level, survey year, alcohol consumption status, smoking status, BMI (continuous), and physical activity. Linear trends across categories were tested using the median values of serum 25(OH)D levels within categories as a continuous variable. Additionally, sensitivity analyses stratified by alcohol consumption status (nondrinkers, moderate drinkers) were conducted to examine associations between serum 25(OH)D levels and elevated serum liver enzymes and NAFLD indices.

Furthermore, stratified analyses by obesity were conducted to examine whether the associations differ according to obesity (i.e., the effect modifier). Likelihood ratio test was performed to verify the effect modification.

We also applied restricted cubic smoothing splines with five knots at the 10th, 25th, 50th, 75th, and 90th centiles to evaluate a nonlinearity such as inverted J-shaped associations. The likelihood ratio test was applied to compare two models, i.e., the model with only the linear term and the model with the linear and the cubic spline terms, for tests for nonlinearity.

We performed statistical analyses using SAS survey procedures (version 9.4, SAS Institute Inc., North Carolina, USA) to incorporate the sample weights and to adjust the analyses for the complex sample design of the survey. In addition, we carried out the restricted cubic smoothing spline regression analysis using the ‘rms’ package: Regression Modeling Strategies version 6.0-1 (The Comprehensive R Archive Network: http://cran.r-project.org accessed on 10 August 2021). All statistical tests were two-sided and *p* values < 0.05 were considered significant.

## 3. Results

The number of subjects with elevated serum ALT and GGT, FLI > 60, and HSI > 36 were 6086 (23.3%), 1134 (12.5%), 919 (10.9%), and 5418 (21.9%), respectively. The proportion of suspected NAFLD was higher in older individuals, smokers, obese individuals, and individuals with lower education levels and lower family income levels than their counterparts (Table 1).

Table 2 presented the distributions of serum 25(OH)D, and serum of AST, ALT and GGT. Serum concentration of 25(OH)D in Korean adults was 16.16 ng/mL and geometric means were higher in males (17.17 ng/mL) than in females (15.48 ng/mL). In addition, the serum levels of AST, ALT and GGT were higher in males than in females.

**Figure 2:**
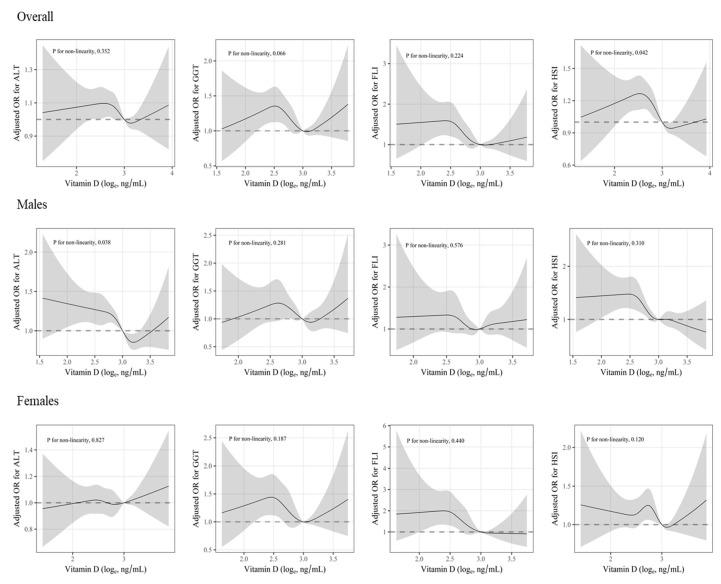
Nonlinear association between serum 25(OH)D levels and risk of suspected NAFLD. A restricted cubic smoothing spline model was applied to allow nonlinear associations. A serum 25(OH)D level of 20 ng/mL was set as the reference. The shaded parts indicate 95% CIs. ORs were adjusted for age, sex, alcohol consumption status (nondrinkers, moderate drinkers), smoking status (never smokers, former smokers, current smokers), level of family income, education level, BMI (continuous, in case of ALT and GGT), physical activity, and survey year.

Associations of serum 25(OH)D levels with elevated serum liver enzymes and NAFLD indices are shown in Table 3. For males, risks of elevated serum ALT and HSI were significantly reduced per unit of natural log-transformed serum 25(OH)D levels (OR [95% (CI)], for ALT, 0.74 [0.60, 0.91], and for HSI, 0.63 [0.50, 0.81]). For females, increase per unit of natural log-transformed serum 25(OH)D levels was associated with the risk of elevated FLI (OR [95% (CI)]: 0.46 [0.27, 0.76]). Similarly, when survey logistic regression models with categorized variables were carried out, significant inverse associations between serum 25(OH)D levels and elevated serum ALT were shown in the highest quartile group for males (OR [95% CI]: 0.68 [0.55, 0.84]) with a dose–response relationship (*p* for trend < 0.01). For HSI, the ORs [95% CI] of the third and the highest quartile group were 0.70 [0.54, 0.90], and 0.62 [0.48, 0.80], respectively, with a dose–response relationship (*p* for trend < 0.01). On the other hand, significant inverse associations between serum 25(OH)D levels and elevated FLI were found only in the third (OR [95% CI]: 0.57 [0.34, 0.95]) and the highest quartile group (OR [95% CI]: 0.51 [0.30, 0.89]) with significant dose–response relationships (*p* for trend < 0.01) in females. In addition, there were significant nonlinear associations between serum 25(OH)D levels and risk of elevated serum ALT and HSI, as a result of a restricted cubic smoothing spline function (Figure 2).

Stratification analyses showed that the effect of serum 25(OH)D levels on elevated serum liver enzymes and NAFLD indices did not differ whether participants were nondrinkers or moderate drinkers, even though for ALT and HSI in moderate drinkers, the ORs [95% CI] of the highest quartile group were 0.82 [0.70, 0.96] and 0.77 [0.62, 0.95], respectively, compared with the lowest quartile group, with dose–response relationships (*p* for trend: ALT = 0.007, HSI = 0.015). Furthermore, BMI significantly increased the risk of elevated serum liver enzymes and NAFLD indices per l kg/m^2^, regardless of alcohol consumption status (Tables S1 and S2).

The protective effect of serum 25(OH)D levels on elevated serum liver enzymes and NAFLD indices was more pronounced in obese individuals. The ORs [95% CI] of suspected NAFLD changed across the quartiles as follows: for serum ALT, from 1.02 [0.85, 1.23] to 0.72 [0.59, 0.87]; for serum GGT, from 0.79 [0.56, 1.13] to 0.64 [0.44, 0.92]; for FLI, from 0.98 [0.67, 1.44] to 0.70 [0.48, 1.02]; and for HSI, from 0.91 [0.73, 1.14] to 0.65 [0.52, 0.81] with dose–response relationships (all *p* for trend < 0.01). The homogeneity of the ORs by obesity was significant for serum ALT (*p* < 0.01) and FLI (*p*= 0.02). On the other hand, there were no significant associations between serum 25(OH)D levels and risk of suspected NAFLD in individuals with normal weight or overweight (Table 4).

## 4. Discussion

This study aimed to evaluate associations between vitamin D and risk of suspected NAFLD in the absence of significant alcohol consumption. Our results suggest that there are inverse associations between sufficient serum 25(OH)D levels and elevated liver enzymes and NAFLD indices (i.e., suspected NAFLD) with strong exposure–response relationships in obese individuals (BMI ≥ 25.0 kg/m^2^) with alcohol consumption status ≤21 standard drinks/week in males and ≤14 standard drinks/week in females. This study supports the idea that vitamin D sufficiency for public health should be emphasized in order to prevent NAFLD, given the rapid increase in the prevalence of obesity and NAFLD worldwide.

The findings of this study are in line with previous epidemiological studies in the general population. Among 1287 non-pregnant women ≥20 years old years in the U.S. NHANES from 1988 to 1994 [32], women with serum 25(OH)D levels of 24.56–32.78 ng/mL had ORs of 0.66 (95% CI: 0.45, 0.96) for hepatic inflammation, which was defined as unexplained elevated serum ALT level (>31 µ/L) without causes of liver inflammation such as hepatitis B, C, or significant alcohol consumption. Moreover, a study of 6567 males aged more than 19 years in 2010 in Korea reported ORs of 1.41 (95% CI: 1.22, 1.63) for NAFLD in subjects with serum 25(OH)D levels ≥ 13.56 ng/mL when compared to subjects with 25(OH)D levels < 16.88 ng/mL [8]. On the other hand, several hospital-based studies have also suggested that low serum 25(OH)vitamin D levels are associated with NAFLD risk, independent of confounders (e.g., age, sex, obesity, and insulin resistance) [23,26].

However, there is limited evidence on associations between serum 25(OH)D levels and risk of NAFLD in obese individuals. In the present study of 25,755 subjects aged equal to or more than 19 years from 2008 to 2014, serum levels of 25(OH)D > 21.40 ng/mL were inversely associated with unexplained elevation of liver enzymes (i.e., ALT > 30 IU/L for males and >19 IU/L for females, and GGT > 51 IU/L for males and >33 IU/L for females) and the NAFLD indices, when compared to subjects with 25(OH)D levels < 13.31 ng/mL.

Worldwide, vitamin D deficiency is prevalent. The prevalence of vitamin D deficiency in general populations is approximately 36% in the United States, 61% in Canada, 92% in Northern Europe, 45–98% in Asia, 31% in Australia and 56% in New Zealand [1]. In the Republic of Korea, the percentage of adults with severe vitamin D deficiency as defined by 25(OH)D < 10 ng/mL has accelerated from 8.63% in 2008 to 16.30% in 2014. More adults have been found to be deficient in vitamin D (i.e., 10 ≤ 25(OH)D < 20 ng/mL), and the percentage has increased from 51.89% to 61.76%. Furthermore, the percentage of adults meeting vitamin D sufficiency as defined by serum 25(OH)D ≥ 30 ng/mL have declined from 8.40% in 2008 to 3.24% in 2014 (Table S3). Although not as serious as Korea, the situation in the U.S. is similar to that of Korea. The prevalence of serum 25(OH)D < 10 ng/mL has increased from 2% in 1988–1994 to 6% in 2001–2004, and serum 25(OH)D ≥ 30 ng/mL has decreased from 45% in 1988–1994 to 23% in 2001–2004 [2].

Recently, the role of vitamin D in the development of liver disease has become a public health concern. In particular, its importance has been strengthened due to vitamin D deficiencies and the prevalence of NAFLD worldwide. Vitamin D is metabolized to 25(OH)D in the liver after intestinal absorption [7]. The mechanism underlying the association between vitamin D and liver damage under obesity is not yet fully elucidated. 25(OH)D acts on adipocytes via vitamin D receptors (VDRs) to inhibit nuclear factor κ-β (NF-κB) transcription, and then inhibits the expression of inflammatory cytokines (e.g., IL -6, TNF-α, TFG-β IL-1β). It also improves inflammation by downregulating the expression of TLR-2, TLR-4, and TLR-9 in Kupffer cells. Furthermore, 25(OH)2D binds to the VDR and acts on hepatic stellate cells and thus ameliorates the proliferation of these cells and production of collagen, which plays a crucial role in the induction of fibrosis through anti-oxidative stress and insulin-sensitizing activities [33,34,35,36]. In addition, previous studies have reported that low serum 25(OH)D levels may reduce the rate of sustained virological response (SVR) under interferon-alfa therapy in patients with hepatitis C virus (HCV) [37,38]. Moreover, 25(OH)D regulates the metabolism of free fatty acids (FFAs) through its direct action on peroxisome proliferator-activated receptor gamma (PPAR-g), increasing FFA-caused insulin resistance in vitro. Thus, the elevation of FFAs due to vitamin D deficiency may lead to fat accumulation into the liver and advance the development of NAFLD. Serum vitamin D may take an active part in both NAFLD and liver cirrhosis Moreover, under conditions of obesity, the active form of vitamin D through 25-hydroxylation and 1α-hydroxylation might be impaired, indicating that adipose tissue changes its metabolism in obesity and passively accumulates vitamin D [39,40]. Further studies on the mechanism of vitamin D to protect the liver from being damaged are needed, especially under conditions of obesity.

There are several limitations in this study. The first is that determining causal associations between serum 25(OH)D levels and elevated liver enzymes and NAFLD indices is not possible with the cross-sectional design of the KNHANES. We would need further prospective studies in order to validate our findings. The second is that the missing rate for serum 25(OH)D in this study was quite high in 2013–2014, which could result in losing representativeness of the whole population and lead to an observation bias. Nevertheless, we used random extraction for the subjects of the serum 25(OH)D test in KNHANES 2013–2014, by survey district, sex, and age (10 s, 20 s, 30 s, 40 s, 50 s, 60 s or older). Thus, representativeness probably has been secured, although concern on observation bias may still remain. Thirdly, the possibility for a batch effect exists, since serum 25(OH)D levels were measured every year from the 2008 to 2014 surveys. We included the survey year in the model so as to overcome this limitation. Even so, a batch effect may still not have been controlled. Fourth, in this study, we were unable to apply an established definition of NAFLD, i.e., evidence of hepatic steatosis (HS) on imaging or from histology in the absence of secondary causes of hepatic fat accumulation. However, unexplained elevation of liver enzymes and NAFLD indices such as HSI or FLI were applied as proxy indicators for NAFLD in previous studies [21,29]. Fifth, the co-effects of other nutrients on NAFLD could not be evaluated. This is demonstrated by recent studies that vitamin A, E, and folate may be inversely associated with the risk of NAFLD [41,42,43]. We would need further studies to investigate the co-effects of nutrients on NAFLD. Sixth, several potentially confounding factors are involved in our study, as with most observational studies. Hence, we made adjustments for age, sex, level of family income, education level, alcohol consumption, smoking status, BMI, and physical activity. Yet, this limitation still remains. Lastly, secondary causes of NAFLD, such as long-term use of a steatogenic medication or monogenic hereditary disorders, could not be considered in this study, because detailed medical history was not available in KNHANES.

This study suggests that obese people are more susceptible to liver damage induced by vitamin D deficiency and that it is necessary to maintain optimal serum vitamin D levels in this population. The best way to get vitamin D is to get enough sunlight so that our body can produce enough vitamin D. Food sources of vitamin D are fatty fish such as salmon, mackerel and tuna etc., egg yolks, cheese, beef liver, mushrooms, etc. In addition, despite inconsistent results, vitamin D supplementation may be necessary in obese populations, regarding reducing the risk of skeletal and extra-skeletal diseases [44].

## 5. Conclusions

In conclusion, 25(OH)D serum levels are inversely associated with the risk of suspected NAFLD in the general population, which is especially pronounced in the obese population. The findings from this study suggest intake of vitamin D for public health should be emphasized in order to prevent adverse health outcomes in obese populations.

## Figures and Tables

**Table 1:** Demographic characteristics of subjects eligible for the study, KNHANES 2008–2014.

	Overall	Elevated Serum ALT ^§^			Elevated Serum GGT ^†,§^			FLI > 60 ^†,§^			HSI > 36 ^§^		
		Yes, N (%)	No, N (%)	*p*	Yes, N (%)	No, N (%)	*p*	Yes, N (%)	No, N (%)	*p*	Yes, N (%)	No, N (%)	*p*
	25,755	6086 (23.3%)	19,669 (76.71%)		1134 (12.5%)	7788 (87.5%)		919 (10.9%)	8003 (89.1%)		5418 (21.9%)	20,337 (78.1%)	
Sex													
Males	9153 (41.76)	1940 (40.21)	7213 (42.23)	0.04	543 (54.25)	2601 (38.94)	<0.01	518 (62.82)	2626 (38.17)	<0.01	2068 (48.21)	7085 (39.95)	<0.01
Females	16,602 (58.24)	4146 (59.79)	12,456 (57.77)		591 (45.75)	5187 (61.06)		401 (37.18)	5377 (61.83)		3350 (51.79)	13,252 (60.05)	
Age, years													
Mean ± SE	45.20 ± 0.16	47.73 ± 0.25	44.43 ± 0.18	<0.01	50.44 ± 0.57	45.07 ± 0.33	<0.01	48.72 ± 0.67	45.37 ± 0.33	<0.01	46.33 ± 0.28	44.88 ± 0.18	<0.01
19–29	3341 (19.89)	486 (13.26)	2855 (21.90)	<0.01	31 (5.56)	974 (21.07)	<0.01	44 (8.83)	961 (20.39)	<0.01	478 (14.68)	2863 (21.35)	<0.01
30–39	4768 (19.96)	937 (18.22)	3831 (20.49)		141 (16.75)	1484 (20.16)		138 (20.04)	1487 (19.70)		942 (20.22)	3826 (19.89)	
40–49	4695 (21.24)	1035 (20.75)	3660 (21.39)		206 (24.73)	1380 (21.17)		178 (24.91)	1408 (21.21)		1064 (23.61)	3631 (20.58)	
50–60	4745 (17.78)	1527 (24.21)	3218 (15.83)		315 (27.88)	1429 (16.90)		202 (21.56)	1542 (17.87)		1193 (20.21)	3552 (17.10)	
60+	8206 (21.12)	2101 (23.56)	6105 (20.38)		441 (25.08)	2521 (20.70)		357 (24.65)	2605 (20.83)		1741 (21.28)	6465 (21.08)	
Smoking status													
Never smokers	17,843 (66.23)	4372 (66.03)	13,471 (66.29)	0.07	644 (51.62)	5491 (67.44)	<0.01	449 (44.64)	5686 (68.00)	<0.01	3669 (61.63)	14,174 (67.52)	<0.01
Former smokers	4014 (15.24)	812 (14.30)	3202 (15.52)		231 (19.60)	1241 (15.27)		229 (23.46)	1243 (14.88)		825 (16.05)	3189 (15.01)	
Current smokers	3898 (18.53)	902 (19.66)	2996 (18.19)		259 (28.77)	1056 (17.29)		241 (31.90)	1074 (17.12)		924 (22.32)	2974 (17.47)	
Alcohol consumption													
Moderate drinkers *	17,286 (72.06)	3841 (67.35)	13,445 (73.49)	<0.01	802 (75.55)	5283 (72.55)	0.09	652 (75.34)	5433 (72.63)	0.18	3462 (68.71)	13,824 (73.00)	<0.01
Nondrinkers	8469 (27.94)	2245 (32.65)	6224 (26.51)		332 (24.45)	2505 (27.45)		267 (24.66)	2570 (27.37)		1956 (31.29)	6513 (27.00)	
Physical activities (≥5 days/week)													
Yes	1872 (8.32)	415 (7.55)	1457 (8.56)	0.06	65 (6.41)	442 (6.08)	0.73	62 (8.01)	445 (5.89)	0.06	366 (7.44)	1506 (8.57)	0.06
No	23,883 (91.68)	5671 (92.45)	18,212 (91.44)		1069 (93.59)	7346 (93.92)		857 (91.99)	7558 (94.11)		5052 (92.56)	18,831 (91.43)	
Family income													
Highest quartile	7090 (28.88)	1603 (27.61)	5487 (29.27)	0.03	270 (24.81)	2125 (27.23)	0.03	200 (24.22)	2195 (27.26)	0.06	1320 (26.13)	5770 (29.65)	<0.01
Third quartile	7057 (29.28)	1645 (28.58)	5412 (29.49)		293 (26.31)	2158 (29.27)		252 (26.68)	2199 (29.17)		1506 (29.68)	5551 (29.16)	
Second quartile	6523 (26.30)	1549 (26.84)	4974 (26.13)		302 (28.25)	1959 (26.63)		253 (30.85)	2008 (26.34)		1448 (28.06)	5075 (25.80)	
Lowest quartile	5085 (15.55)	1289 (16.96)	3796 (15.12)		269 (20.63)	1546 (16.87)		214 (18.25)	1601 (17.23)		1144 (16.12)	3941 (15.38)	
Education level													
Elementary school or less	6882 (18.96)	1992 (24.24)	4890 (17.35)	<0.01	365 (25.37)	2016 (19.64)	<0.01	295 (25.52)	2086 (19.72)	<0.01	1680 (21.72)	5202 (18.18)	<0.01
Middle school	2760 (9.84)	784 (12.31)	1976 (9.09)		171 (14.80)	807 (9.56)		130 (12.51)	848 (9.93)		655 (11.31)	2105 (9.43)	
High school	8621 (38.40)	1829 (35.04)	6792 (39.43)		368 (37.07)	2519 (36.72)		265 (33.08)	2622 (37.21)		1715 (36.37)	6906 (38.97)	
College or more	7492 (32.80)	1481 (28.42)	6011 (34.13)		230 (22.75)	2446 (34.08)		229 (28.89)	2447 (33.13)		1368 (30.60)	6124 (33.42)	
BMI (kg/m^2^)													
Mean ± SE	23.51 ± 0.03	25.43 ± 0.07	22.93 ± 0.03	<0.01	25.25 ± 0.14	23.21 ± 0.06	<0.01	28.51 ± 0.14	22.85 ± 0.05	<0.01	27.73 ± 0.06	22.33 ± 0.02	<0.01
< 23 kg/m^2^	11,952 (46.91)	1554 (25.56)	10,398 (53.39)	<0.01	296 (25.04)	3897 (50.70)	<0.01	19 (1.65)	4174 (53.09)	<0.01	160 (3.32)	11,792 (59.14)	<0.01
23–24.9 kg/m^2^	6000 (23.10)	1396 (22.43)	4604 (23.30)		281 (23.14)	1726 (22.12)		91 (9.56)	1916 (23.79)		643 (11.92)	5357 (26.24)	
≥ 25 kg/m^2^	7803 (29.99)	3136 (52.01)	4667 (23.30)		557 (51.82)	2165 (27.18)		809 (88.79)	1913 (23.13)		4615 (84.76)	3188 (14.63)	
Survey year													
2008	4326 (12.68)	988 (12.29)	3338 (12.80)	0.84	-	-	0.46	-	-	0.72	930 (12.77)	3396 (12.66)	0.11
2009	5412 (14.88)	1302 (15.26)	4110 (14.76)		-	-		-	-		1179 (15.02)	4233 (14.83)	
2010	4379 (14.35)	1022 (14.24)	3357 (14.38)		546 (46.86)	3833 (48.39)		451 (47.48)	3928 (48.29)		860 (13.48)	3519 (14.60)	
2011	4543 (15.42)	1103 (15.92)	3440 (15.27)		588 (53.14)	3955 (51.61)		468 (52.52)	4075 (51.71)		950 (15.52)	3593 (15.39)	
2012	4209 (14.99)	981 (14.53)	3228 (15.13)		-	-		-	-		861 (14.68)	3348 (15.08)	
2013	1478 (17.17)	349 (17.53)	1129 (17.06)		-	-		-	-		341 (18.80)	1137 (16.71)	
2014	1408 (10.51)	341 (10.23)	1067 (10.60)		-	-		-	-		297 (9.74)	1111 (10.73)	
25(OH)D (ng/mL) GM ± SE	16.16 ± 0.09	16.18 ± 0.13	16.16 ± 0.10	0.83	16.81 ± 0.28	16.29 ± 0.17	0.04	16.57 ± 0.29	16.33 ± 0.17	0.37	16.0 ± 0.13	16.20 ± 0.10	0.16

* ≤21 drinks /week in males, ≤14 drinks /week in females; ^†^ Information on data for GGT and FLI is presented separately, hence serum GGT was measured only in 2010~2011. ^§^ Elevated serum ALT was defined as >30 IU/L for males and >19 IU/L for females. Elevated serum GGT was defined as >51 IU/L for males and >33 IU/L for females. NAFLD was defined as FLI > 60 and HSI > 36. FLI: fatty liver index, HSI: hepatic steatosis index, BMI: body mass index, GM: geometric mean, SE: standard error.

**Table 2:** Distribution of serum 25(OH)D levels and serum liver enzymes in the study population KNHANES 2008–2014.

		N (%)	GM (95% CI)	Min	10%	25th	Median	75_th_	90%	Max
25(OH)D (ng/mL)										
	Males	9153 (41.76)	17.17 (16.94, 17.40)	3.01	11.21	14.25	18.29	23.02	28.13	60.47
	Females	16,602 (58.24)	15.48 (15.29, 15.67)	1.98	9.96	12.45	15.95	20.44	25.70	66.96
	Overall	25,755	16.16 (15.98, 16.35)	1.98	10.29	13.01	16.77	21.40	26.72	66.96
25(OH)D * (ng/mL)										
	Males	3144 (40.85)	17.51 (17.10, 17.92)	4.08	11.55	14.36	18.24	22.69	27.40	48.82
	Females	5778 (59.15)	15.60 (15.28, 15.94)	4.11	10.16	12.52	15.89	20.03	24.83	51.44
	Overall	8922	16.36 (16.02, 16.69)	4.08	10.49	13.13	16.74	21.05	25.99	51.44
AST (IU/L)										
	Males	9153 (41.76)	21.24 (21.05, 21.43)	7.00	15.00	18.00	21.00	25.00	32.00	323.00
	Females	16,602 (58.24)	18.59 (18.48, 18.71)	5.00	14.00	16.00	18.00	22.00	27.00	201.00
	Overall	25,755	19.66 (19.55, 19.77)	5.00	14.00	16.00	19.00	23.00	29.00	323.00
ALT (IU/L)										
	Males	9153 (41.76)	21.56 (21.26, 21.86)	4.00	12.00	15.00	20.00	28.00	41.00	293.00
	Females	16,602 (58.24)	14.90 (14.75, 15.06)	2.00	9.00	11.00	14.00	19.00	27.00	393.00
	Overall	25,755	17.39 (17.23, 17.55)	2.00	10.00	12.00	16.00	23.00	33.00	393.00
GGT (IU/L) *										
	Males	3144 (40.85)	29.25 (28.44, 30.09)	9.00	15.00	19.00	26.00	41.00	68.00	1381.00
	Females	5778 (59.15)	17.31 (17.00, 17.62)	4.00	11.00	13.00	16.00	23.00	34.00	662.00
	Overall	8922	21.45 (21.08, 21.82)	4.00	11.00	14.00	19.00	29.00	47.00	1381.00

GM: geometric mean. * Information on data for GGT and FLI is presented separately, hence serum GGT was measured only in 2010~2011.

**Table 3:** Association between serum 25(OH)D levels, elevated serum liver enzymes, and NAFLD indices.

		Overall			Males			Females		
ALT ^§^	Concentration (ng/mL)	Yes	OR * (95% CI)	OR ** (95% CI)	Yes	OR * (95% CI)	OR ** (95% CI)	Yes	OR * (95% CI)	OR ** (95% CI)
		n = 6086			n = 1940			n = 4146		
25(OH)D, continuous			0.91 (0.81, 1.02)	0.95 (0.84, 1.07)		0.74 (0.61, 0.89)	0.74 (0.60, 0.91)		1.05 (0.91, 1.21)	1.10 (0.95, 1.28)
1st quartile	< 13.01	1494 (26.53)	Reference	Reference	403 (24.12)	Reference	Reference	1091 (28.16)	Reference	Reference
2nd quartile	13.01–16.77	1558 (26.67)	1.08 (0.96, 1.20)	1.02 (0.90, 1.14)	486 (26.06)	0.95 (0.78, 1.16)	0.86 (0.70, 1.06)	1072 (27.08)	1.14 (1.00, 1.31)	1.11 (0.96, 1.27)
3rd quartile	16.77–21.40	1541 (25.23)	1.02 (0.91, 1.14)	1.02 (0.90, 1.14)	551 (28.16)	0.93 (0.76, 1.13)	0.91 (0.73, 1.12)	990 (23.26)	1.06 (0.92, 1.22)	1.06 (0.92, 1.23)
4th quartile	>21.40	1493 (21.56)	0.88 (0.78, 0.99)	0.91 (0.80, 1.03)	500 (21.66)	0.69 (0.57, 0.84)	0.68 (0.55, 0.84)	993 (21.50)	1.07 (0.92, 1.23)	1.11 (0.95, 1.30)
*p* for trend			0.01	0.13		<0.01	<0.01		0.58	0.24
GGT ^†^	Concentration (ng/mL)	Yes	OR * (95% CI)	OR ** (95% CI)	Yes	OR * (95% CI)	OR ** (95% CI)	Yes	OR * (95% CI)	OR ** (95% CI)
		n = 1134			n = 543			n = 591		
25(OH)D, continuous			0.90 (0.71, 1.14)	0.90 (0.71, 1.15)		0.98 (0.66, 1.43)	0.98 (0.66, 1.45)		0.82 (0.61, 1.11)	0.83 (0.61, 1.14)
1st quartile	< 13.01	261 (24.34)	Reference	Reference	92 (20.01)	Reference	Reference	169 (29.48)	Reference	Reference
2nd quartile	13.01–16.77	295 (26.09)	0.93 (0.75, 1.16)	0.87 (0.69, 1.09)	125 (22.23)	0.80 (0.56, 1.16)	0.72 (0.50, 1.04)	170 (30.66)	1.03 (0.79, 1.36)	1.00 (0.76, 1.32)
3rd quartile	16.77–21.40	294 (24.90)	0.78 (0.63, 0.97)	0.77 (0.62, 0.96)	162 (28.47)	0.81 (0.57, 1.15)	0.76 (0.53, 1.09)	132 (20.66)	0.73 (0.54, 0.99)	0.74 (0.55, 1.00)
4th quartile	> 21.40	284 (24.67)	0.84 (0.65, 1.07)	0.84 (0.66, 1.08)	164 (29.29)	0.85 (0.58, 1.23)	0.84 (0.57, 1.22)	120 (19.20)	0.81 (0.59, 1.12)	0.82 (0.59, 1.14)
*p* for trend			0.11	0.17		0.56	0.69		0.07	0.10
FLI ^†,§^	Concentration (ng/mL)	Yes	OR * (95% CI)	OR ** (95% CI)	Yes	OR * (95% CI)	OR ** (95% CI)	Yes	OR * (95% CI)	OR ** (95% CI)
		n = 919			n = 518			n = 401		
25(OH)D, continuous			0.76 (0.58, 0.99)	0.70 (0.49, 1.01)		0.90 (0.64, 1.26)	0.90 (0.57, 1.41)		0.60 (0.41, 0.86)	0.46 (0.27, 0.76)
1st quartile	< 13.01	209 (23.18)	Reference	Reference	84 (18.42)	Reference	Reference	125 (31.22)	Reference	Reference
2nd quartile	13.01–16.77	265 (30.06)	1.13 (0.87, 1.48)	0.87 (0.62, 1.23)	139 (28.29)	1.19 (0.81, 1.77)	0.72 (0.45, 1.14)	126 (33.04)	1.05 (0.75, 1.47)	1.03 (0.64, 1.66)
3rd quartile	16.77–21.40	224 (24.40)	0.78 (0.59, 1.02)	0.66 (0.46, 0.94)	145 (27.88)	0.92 (0.63, 1.35)	0.68 (0.43, 1.09)	79 (18.51)	0.61 (0.42, 0.89)	0.57 (0.34, 0.95)
4th quartile	>21.40	221 (22.37)	0.76 (0.57, 1.01)	0.72 (0.51, 1.03)	150 (25.41)	0.86 (0.59, 1.26)	0.83 (0.52, 1.31)	71 (17.22)	0.67 (0.43, 1.03)	0.51 (0.30, 0.89)
*p* for trend			<0.01	0.04		0.16	0.71		0.01	<0.01
HSI ^§^	Concentration (ng/mL)	Yes	OR * (95% CI)	OR ** (95% CI)	Yes	OR * (95% CI)	OR ** (95% CI)	Yes	OR * (95% CI)	OR ** (95% CI)
		n = 5418			n = 2068			n = 3350		
25(OH)D, continuous			0.81 (0.72, 0.90)	0.79 (0.67, 0.92)		0.71 (0.60, 0.85)	0.63 (0.50, 0.81)		0.91 (0.79, 1.04)	0.97 (0.78, 1.21)
1st quartile	<13.01	1315 (26.74)	Reference	Reference	428 (24.62)	Reference	Reference	887 (28.71)	Reference	Reference
2nd quartile	13.01–16.77	1459 (27.69)	1.09 (0.97, 1.23)	0.96 (0.81, 1.13)	522 (26.64)	0.96 (0.80, 1.16)	0.78 (0.60, 1.00)	937 (28.66)	1.20 (1.04, 1.38)	1.12 (0.91, 1.38)
3rd quartile	16.77–21.40	1371 (24.87)	0.95 (0.85, 1.07)	0.91 (0.78, 1.07)	563 (26.04)	0.82 (0.68, 0.99)	0.70 (0.54, 0.90)	808 (23.79)	1.07 (0.92, 1.25)	1.18 (0.95, 1.46)
4th quartile	>21.40	1273 (20.70)	0.81 (0.72, 0.91)	0.77 (0.65, 0.91)	555 (22.69)	0.73 (0.60, 0.88)	0.62 (0.48, 0.80)	718 (18.84)	0.90 (0.77, 1.05)	0.93 (0.75, 1.17)
*p* for trend			<0.01	<0.01		<0.01	<0.01		0.09	0.65

* Odds ratios (ORs) adjusted for age (continuous) and sex (in case of overall). ** ORs further adjusted for alcohol consumption status (nondrinkers, moderate drinkers), smoking status (never, former, current), level of family income, education level, BMI (continuous, in case of ALT and GGT), physical activity, and survey year. ^†^ Measured only in 2010~2011. ^§^ Elevated serum ALT was defined as >30 IU/L for males and >19 IU/L for females. Elevated serum GGT was defined as >51 IU/L for males and >33 IU/L for females. NAFLD was defined as FLI > 60 and HSI >36. FLI: fatty liver index, HSI: hepatic steatosis index.

**Table 4:** Associations between serum 25(OH)D levels, elevated serum liver enzymes, and NAFLD indices, stratified by obesity.

		Obesity									Effect modification ***
		BMI < 23 kg/m^2^			BMI: 23–24.9 kg/m^2^			BMI ≥ 25 kg/m^2^			
ALT ^§^	Concentration (ng/mL)	Yes	OR * (95% CI)	OR ** (95% CI)	Yes	OR * (95% CI)	OR ** (95% CI)	Yes	OR * (95% CI)	OR ** (95% CI)	
		n = 1554			n = 1396			n = 3136			
25(OH)D, continuous			1.15 (0.93, 1.41)	1.16 (0.94, 1.42)		0.90 (0.71, 1.14)	0.90 (0.71, 1.14)		0.76 (0.63, 0.90)	0.80 (0.67, 0.96)	
1st quartile	<13.01	413 (27.41)	Reference	Reference	339 (26.80)	Reference	Reference	742 (25.99)	Reference	Reference	<0.01
2nd quartile	13.01–16.77	378 (25.50)	1.10 (0.90, 1.35)	1.10 (0.90, 1.35)	342 (24.21)	0.90 (0.71, 1.13)	0.90 (0.71, 1.13)	838 (28.31)	1.05 (0.88, 1.26)	1.02 (0.85, 1.23)	
3rd quartile	16.77–21.40	355 (23.39)	1.04 (0.84, 1.28)	1.04 (0.84, 1.30)	348 (23.55)	0.83 (0.65, 1.05)	0.83 (0.65, 1.06)	838 (26.86)	1.04 (0.87, 1.25)	1.09 (0.90, 1.31)	
4th quartile	>21.40	408 (23.69)	1.11 (0.89, 1.38)	1.13 (0.91, 1.41)	367 (25.44)	0.96 (0.77, 1.21)	0.96 (0.76, 1.22)	718 (18.84)	0.69 (0.57, 0.83)	0.72 (0.59, 0.87)	
*p* for trend			0.46	0.35		0.76	0.75		<0.01	<0.01	
GGT ^†,§^	Concentration (ng/mL)	Yes	OR * (95% CI)	OR ** (95% CI)	Yes	OR * (95% CI)	OR ** (95% CI)	Yes	OR * (95% CI)	OR ** (95% CI)	
		n = 296			n = 281			n = 557			
25(OH)D, continuous			1.11 (0.70, 1.78)	1.12 (0.70, 1.77)		1.08 (0.69, 1.69)	1.09 (0.69, 1.72)		0.70 (0.49, 1.00)	0.70 (0.49, 0.99)	
1st quartile	<13.01	72 (24.57)	Reference	Reference	54 (20.83)	Reference	Reference	135 (25.80)	Reference	Reference	0.06
2nd quartile	13.01–16.77	68 (21.60)	0.87 (0.55, 1.37)	0.87 (0.55, 1.39)	70 (24.62)	1.03 (0.66, 1.61)	1.07 (0.67, 1.70)	157 (28.91)	0.80 (0.56, 1.13)	0.79 (0.56, 1.13)	
3^rd^ quartile	16.77–21.40	74 (25.62)	0.85 (0.55, 1.32)	0.84 (0.55, 1.30)	75 (24.90)	0.91 (0.59, 1.43)	0.92 (0.58, 1.45)	145 (24.55)	0.66 (0.47, 0.93)	0.66 (0.47, 0.92)	
4th quartile	>21.40	82 (28.21)	1.04 (0.65, 1.68)	1.05 (0.66, 1.68)	82 (29.66)	1.06 (0.65, 1.71)	1.07 (0.66, 1.73)	120 (20.74)	0.63 (0.44, 0.91)	0.64 (0.44, 0.92)	
*p* for trend			0.79	0.78		0.88	0.87		<0.01	0.01	
FLI ^†,§^	Concentration (ng/mL)	Yes	OR * (95% CI)	OR ** (95% CI)	Yes	OR * (95% CI)	OR ** (95% CI)	Yes	OR * (95% CI)	OR ** (95% CI)	
		n = 19			n = 91			n = 809			
25(OH)D, continuous			1.10 (0.20, 6.00)	0.92 (0.15, 5.46)		0.72 (0.34, 1.51)	0.75 (0.36, 1.60)		0.67 (0.48, 0.94)	0.68 (0.47, 1.01)	
1st quartile	< 13.01	6 (28.78)	Reference	Reference	20 (23.99)	Reference	Reference	183 (22.99)	Reference	Reference	0.02
2nd quartile	13.01–16.77	1 (3.86)	0.12 (0.01, 1.16)	0.08 (0.01, 0.90)	18 (17.66)	0.57 (0.26, 1.22)	0.59 (0.26, 1.33)	246 (31.88)	1.04 (0.77, 1.41)	0.98 (0.67, 1.44)	
3rd quartile	16.77–21.40	4 (28.58)	0.59 (0.13, 2.74)	0.43 (0.10, 1.88)	22 (23.25)	0.62 (0.30, 1.28)	0.61 (0.29, 1.29)	198 (24.44)	0.70 (0.51, 0.97)	0.68 (0.46, 1.00)	
4th quartile	> 21.40	8 (38.79)	0.75 (0.21, 2.72)	0.55 (0.15, 2.00)	31 (35.10)	0.79 (0.37, 1.68)	0.83 (0.40, 1.73)	182 (20.69)	0.68 (0.49, 0.95)	0.70 (0.48, 1.02)	
*p* for trend			0.93	0.91		0.85	0.93		<0.01	0.02	
HSI ^§^	Concentration (ng/mL)	Yes	OR * (95% CI)	OR ** (95% CI)	Yes	OR * (95% CI)	OR ** (95% CI)	Yes	OR * (95% CI)	OR ** (95% CI)	
		n = 160			n = 643			n = 4615			
25(OH)D, continuous			0.93 (0.58, 1.48)	0.90 (0.52, 1.55)		0.95 (0.71, 1.27)	0.94 (0.70, 1.27)		0.63 (0.53, 0.76)	0.70 (0.56, 0.87)	
1st quartile	< 13.01	45 (25.82)	Reference	Reference	159 (25.47)	Reference	Reference	1111 (26.96)	Reference	Reference	0.13
2nd quartile	13.01–16.77	44 (25.75)	1.12 (0.66, 1.90)	1.05 (0.60, 1.83)	159 (25.47)	0.97 (0.72, 1.30)	0.99 (0.73, 1.34)	1256 (28.08)	0.96 (0.79, 1.16)	0.91 (0.73, 1.14)	
3rd quartile	16.77–21.40	41 (31.46)	1.40 (0.77, 2.53)	1.32 (0.69, 2.50)	164 (23.37)	0.84 (0.62, 1.13)	0.84 (0.63, 1.14)	1166 (24.83)	0.79 (0.65, 0.96)	0.88 (0.71, 1.10)	
4th quartile	> 21.40	30 (16.97)	0.81 (0.44, 1.49)	0.83 (0.44, 1.58)	161 (25.68)	1.02 (0.75, 1.39)	1.04 (0.76, 1.43)	1082 (20.14)	0.63 (0.52, 0.75)	0.65 (0.52, 0.81)	
*p* for trend			0.64	0.75		0.99	0.94		<0.01	<0.01	

* Odds ratio (OR) adjusted for age (continuous) and sex (in case of overall). ** OR further adjusted for alcohol consumption status (nondrinkers, moderate drinkers), smoking status (continuous, pack-years), level of family income, education level, BMI (continuous, in case of ALT and GGT), physical activity, and survey year. *** likelihood ratio test. ^†^ Measured only in 2010~2011. ^§^ Elevated serum ALT was defined as >30 IU/L for males and >19 IU/L for females. Elevated serum GGT was defined as >51 IU/L for males and >33 IU/L for females. NAFLD was defined as FLI > 60 and HSI > 36.

## Data Availability

Data described in the manuscript and code book will be made publicly and freely available without restriction at https://knhanes.cdc.go.kr/knhanes/sub03/sub03_02_05.do accessed on 10 August 2021. However, analytic codes will be made available upon request pending application.

## Supplementary Materials

The following are available online at https://www.mdpi.com/article/10.3390/ijerph18168682/s1, Table S1. Stratification analysis by alcohol consumption (ALT, NAFLD-HSI); Table S2. Stratification analysis by alcohol consumption (GGT, NAFLD-FLI); Table S3. Distribution of serum vitamin D concentrations by year, KNHANES 2008-2014.

## Abbreviations

25(OH)D: 25-hydroxyvitamin D, ALT: alanine aminotransferase, AST: aspartate aminotransferase, BMI: body mass index, CI: confidence interval, FLI: fatty liver index, GGT: gamma-glutamyl transferase, GM: geometric mean, SE: standard error, HBV: hepatitis B virus, HCV: hepatitis C virus, HSI; hepatic steatosis index, IARC: International Agency for Research on Cancer, KCDC: Korea Centers for Disease Control and Prevention, KNHANES: Korea National Health and Nutrition Examination Survey, NAFLD: non-alcoholic fatty liver disease, NHANES: National Health and Nutrition Examination Survey, OR: odds ratio
