# Supplementary material for: Inverse Association between Serum 25-hydroxyvitamin D Levels and Risk of Suspected Non-Alcoholic Fatty Liver Disease in Obese Population
Source: Int J Environ Res Public Health. 2021 Aug 17;18(16):8682. doi: 10.3390/ijerph18168682 (PMC8394297; doi:10.3390/ijerph18168682)
Supplement: Supplementary file 1 [file ijerph-18-08682-s001.zip › ijerph-1318038-supplementary.pdf]

Table S1. Stratification analysis by alcohol consumption (ALT, NAFLD-HSI)

	Alcohol consumption									
	Non-drinkers					Moderate drinkers				
	ALT			NAFLD		ALT			NAFLD	
		Elevation of ALT (%)	OR	HSI (%)	OR		Elevation of ALT (%)	OR	HSI (%)	OR
Vitamin D										
1st quartile	2196 (28.68)	583 (27.93)	Reference	514 (29.15)	Reference	4234 (26.75)	911 (25.86)	Reference	801 (25.64)	Reference
2nd quartile	2056 (24.88)	538 (25.24)	1.01 (0.82, 1.23)	529 (28.13)	0.93 (0.71, 1.23)	4385 (25.91)	1020 (27.37)	1.02 (0.88, 1.17)	930 (27.48)	0.97 (0.79, 1.18)
3rd quartile	2001 (23.03)	519 (22.77)	1.05 (0.86, 1.29)	437 (20.60)	0.78 (0.57, 1.07)	4443 (25.33)	1022 (26.42)	0.99 (0.86, 1.14)	934 (26.82)	0.97 (0.81, 1.17)
4th quartile	2216 (23.41)	605 (24.06)	1.09 (0.88, 1.34)	476 (22.12)	0.76 (0.57, 1.00)	4224 (22.01)	888 (20.35)	0.82 (0.70, 0.96)	797 (20.05)	0.77 (0.62, 0.95)
P for tend			0.3818		0.0344			0.0073		0.0151
Age										
Mean±SE	52.41±0.26	53.99±0.41	1.00 (1.00, 1.01)	53.44±0.46	1.00 (0.99, 1.01)	42.40±0.17	44.70±0.31	1.01 (1.00, 1.01)	43.09±0.32	0.99 (0.98, 0.99)
Sex										
Men	2050 (27.90)	416 (24.82)	Reference	468 (31.57)	Reference	7103 (47.14)	1524 (47.67)	Reference	1600 (55.79)	Reference
Women	6419 (72.10)	1829 (75.18)	1.31 (1.06, 1.62)	1488 (68.43)	0.91 (0.67, 1.24)	10183 (52.86)	2317 (52.33)	1.21 (1.05, 1.38)	1862 (44.21)	0.91 (0.75, 1.11)
BMI (kg/m^2)										
Mean ± SE	23.70±0.05	25.15±0.11	1.19 (1.16, 1.21)	27.71±0.10	2.45 (2.30, 2.61)	23.44±0.04	25.56±0.08	1.27 (1.25, 1.29)	27.74±0.07	2.49 (2.38, 2.59)
Smoking status										
Never smokers	6659 (77.25)	1864 (78.67)	Reference	1548 (75.11)	Reference	11184 (61.95)	2508 (59.91)	Reference	2121 (55.50)	Reference
Former smokers	1109 (13.08)	218 (10.98)	0.87 (0.67, 1.13)	236 (13.24)	0.93 (0.65, 1.35)	2905 (16.08)	594 (15.92)	1.01 (0.85, 1.19)	589 (17.32)	1.03 (0.82, 1.29)
Current smokers	701 (9.67)	163 (10.35)	1.30 (0.98, 1.74)	172 (11.65)	1.66 (1.03, 2.67)	3197 (21.97)	739 (24.17)	1.25 (1.08, 1.45)	752 (27.18)	1.39 (1.12, 1.73)
Education										
Elementary school or less	3639 (32.89)	1048 (36.97)	Reference	918 (35.55)	Reference	3243 (13.56)	944 (18.07)	Reference	762 (15.42)	Reference
Middle school	989 (11.85)	297 (13.90)	1.08 (0.85, 1.36)	244 (13.36)	1.12 (0.81, 1.56)	1771 (9.06)	487 (11.54)	1.02 (0.84, 1.24)	411 (10.38)	1.03 (0.78, 1.35)
High school	2125 (30.07)	523 (27.24)	0.84 (0.67, 1.05)	466 (28.17)	1.07 (0.79, 1.46)	6496 (41.63)	1306 (38.82)	0.79 (0.66, 0.95)	1249 (40.10)	0.86 (0.68, 1.08)
College or more	1716 (25.19	377 (21.90)	0.88 (0.69, 1.13)	328 (22.91)	1.45 (1.01, 2.09)	5776 (35.75)	1104 (31.58)	0.77 (0.63, 0.94)	1040 (34.10)	0.90 (0.69, 1.17)
Family income										
Highest quartile	1753 (22.08)	470 (21.31)	Reference	351 (18.44)	Reference	5337 (31.52)	1133 (30.66)	Reference	969 (29.64)	Reference
Third quartile	1936 (25.30)	527 (26.72)	1.02 (0.82, 1.26)	473 (26.86)	1.04 (0.77, 1.40)	5121 (30.82)	1118 (29.49)	0.91 (0.80, 1.03)	1033 (30.96)	1.02 (0.85, 1.24)
Second quartile	2256 (28.67)	575 (26.71)	0.84 (0.69, 1.04)	519 (28.58)	1.11 (0.83, 1.49)	4267 (25.38)	974 (26.91)	0.93 (0.81, 1.08)	929 (27.83)	0.95 (0.77, 1.16)
Lowest quartile	2524 (23.95)	673 (25.26)	0.88 (0.70, 1.11)	613 (26.12)	1.10 (0.80, 1.53)	2561 (12.29)	616 (12.94)	0.83 (0.69, 1.00)	531 (11.57)	0.68 (0.53, 0.88)
Physical activities (5 days/week)										
Yes	511 (7.01)	128 (5.91)	Reference	109 (6.37)	Reference	1361 (8.83)	287 (8.35)	Reference	257 (7.92)	Reference
No	7958 (92.99)	2117 (94.09)	1.39 (1.02, 1.90)	1847 (93.63)	2.12 (1.43, 3.14)	15925 (91.17)	3554 (91.65)	1.08 (0.90, 1.30)	3205 (92.08)	1.47 (1.15, 1.88)
Survey year										
2008	1544 (13.51)	413 (13.53)	Reference	368 (13.70)	Reference	2782 (12.36)	575 (11.68)	Reference	562 (12.35)	Reference
2009	1842 (15.49)	468 (14.78)	0.90 (0.74, 1.09)	432 (15.30)	0.75 (0.57, 0.98)	3570 (14.64)	834 (15.50)	1.07 (0.92, 1.24)	747 (14.89)	0.81 (0.65, 1.01)
2010	1377 (13.83)	361 (13.60)	0.96 (0.77, 1.19)	295 (12.65)	0.60 (0.46, 0.80)	3002 (14.55)	661 (14.56)	1.03 (0.88, 1.21)	565 (13.85)	0.80 (0.63, 1.01)
2011	1460 (15.02)	400 (15.66)	1.02 (0.82, 1.27)	335 (15.39)	0.82 (0.60, 1.10)	3083 (15.58)	703 (16.04)	1.02 (0.87, 1.20)	615 (15.59)	0.75 (0.59, 0.95)
2012	1457 (15.63)	367 (13.46)	0.78 (0.64, 0.94)	328 (14.58)	0.59 (0.43, 0.80)	2752 (14.74)	614 (15.05)	0.97 (0.84, 1.13)	533 (14.72)	0.66 (0.52, 0.85)
2013	395 (17.05)	116 (18.49)	1.07 (0.79, 1.43)	102 (19.18)	0.91 (0.61, 1.35)	1083 (17.21)	233 (17.06)	0.96 (0.77, 1.20)	239 (18.62)	0.86 (0.65, 1.14)
2014	394 (9.45)	120 (10.48)	1.26 (0.91, 1.74)	96 (9.20)	0.89 (0.58, 1.38)	1014 (10.92)	221 (10.11)	0.92 (0.73, 1.15)	201 (9.98)	0.59 (0.43, 0.81)

ORs adjusted for age, sex, smoking status (never, former, current), family income level, education level, BMI (continuous), physical activity, and survey year.

Table S2. Stratification analysis by alcohol consumption (GGT, NAFLD-FLI)

	Alcohol consumption									
	Non-drinkers					Moderate drinkers				
		GGT		NAFLD			GGT		NAFLD	
		Elevation of GGT (%)	OR	FLI (%)	OR		Elevation of GGT (%)	OR	FLI (%)	OR
Vitamin D										
1st quartile	688 (25.13)	94 (31.46)	Reference	77 (28.54)	Reference	1469 (25.13)	167 (22.04)	Reference	132 (21.42)	Reference
2nd quartile	751 (27.29)	100 (28.92)	0.75 (0.50, 1.12)	79 (32.43)	0.72 (0.38, 1.39)	1574 (26.49)	195 (25.17)	0.95 (0.71, 1.27)	186 (29.28)	0.97 (0.65, 1.44)
3rd quartile	696 (24.51)	66 (17.23)	0.50 (0.32, 0.77)	50 (16.79)	0.52 (0.26, 1.04)	1668 (27.06)	228 (27.38)	0.88 (0.67, 1.16)	174 (26.89)	0.74 (0.49, 1.12)
4th quartile	702 (23.07)	72 (22.39)	0.66 (0.44, 1.00)	61 (22.24)	0.55 (0.29, 1.03)	1374 (21.32)	212 (25.41)	0.93 (0.68, 1.27)	160 (22.41)	0.83 (0.54, 1.25)
P for tend			0.0366		0.0580			0.6368		0.2632
Age										
Mean ± SE	53.70±0.49	55.94±1.02	1.01 (0.99, 1.02)	56.69±1.26	1.03 (1.01, 1.05)	42.78±0.31	48.66±0.61	1.03 (1.02, 1.04)	46.11±0.69	1.02 (1.01, 1.03)
Sex										
Men	652 (26.68)	74 (29.11)	Reference	86 (42.48)	Reference	2492 (46.11)	469 (62.39)	Reference	432 (69.48)	Reference
Women	2185 (73.32)	258 (70.89)	0.70 (0.44, 1.10)	181 (57.52)	0.31 (0.15, 0.63)	3593 (53.89)	333 (37.61)	0.60 (0.45, 0.80)	220 (30.52)	0.21 (0.14, 0.30)
BMI (kg/m^2)										
Mean ± SE	23.66±0.09	25.25±0.23±	1.14 (1.10, 1.19)	28.98±0.26	2.06 (1.86, 2.27)	23.39±0.06	25.25±0.16	1.19 (1.15, 1.22)	28.36±0.16	2.10 (1.98, 2.22)
Smoking status										
Never smokers	2221 (76.14)	269 (76.47)	Reference	186 (61.21)	Reference	3914 (61.50)	375 (43.58)	Reference	263 (39.22)	Reference
Former smokers	395 (14.75)	41 (16.19)	0.85 (0.51, 1.42)	49 (23.08)	1.77 (0.76, 4.15)	1077 (16.21)	190 (20.71)	1.13 (0.82, 1.56)	180 (23.59)	1.53 (0.99, 2.35)
Current smokers	221 (9.11)	22 (7.34)	0.60 (0.29, 1.24)	32 (15.71)	1.62 (0.54, 4.87)	1094 (22.29)	237 (35.71)	2.01 (1.49, 2.71)	209 (37.19)	2.94 (1.95, 4.42)
Education										
Elementary school or less	1241 (36.83)	159 (41.35)	Reference	136 (43.48)	Reference	1140 (14.24)	206 (20.20)	Reference	159 (19.64)	Reference
Middle school	351 (12.20)	46 (13.70)	1.03 (0.65, 1.61)	43 (14.69)	0.94 (0.50, 1.78)	627 (9.47)	125 (15.16)	1.30 (0.94, 1.79)	87 (11.80)	0.65 (0.41, 1.04)
High school	692 (27.45)	81 (27.35)	1.03 (0.63, 1.71)	53 (24.81)	0.88 (0.47, 1.64)	2195 (40.23)	287 (40.22)	1.08 (0.78, 1.48)	212 (35.79)	0.51 (0.31, 0.85)
College or more	553 (23.52)	46 (17.59)	0.80 (0.43, 1.50)	35 (17.02)	0.88 (0.43, 1.80)	2123 (36.07)	184 (24.42)	0.72 (0.50, 1.03)	194 (32.77)	0.53 (0.31, 0.88)
Family income										
Highest quartile	576 (20.37)	63 (17.05)	Reference	47 (18.38)	Reference	1819 (29.37)	207 (27.32)	Reference	153 (26.13)	Reference
Third quartile	643 (23.27)	79 (25.85)	1.28 (0.82, 1.99)	69 (23.45)	0.99 (0.51, 1.93)	1808 (30.99)	214 (26.46)	0.86 (0.67, 1.10)	183 (27.74)	0.84 (0.60, 1.18)
Second quartile	752 (29.96)	87 (27.11)	0.98 (0.61, 1.55)	63 (30.49)	0.77 (0.39, 1.52)	1509 (25.67)	215 (28.62)	1.01 (0.78, 1.32)	190 (30.97)	1.13 (0.78, 1.64)
Lowest quartile	866 (26.41)	103 (29.98)	1.16 (0.66, 2.04)	88 (27.69)	0.68 (0.34, 1.38)	949 (13.97)	166 (17.60)	0.95 (0.70, 1.29)	126 (15.15)	1.02 (0.63, 1.63)
Physical activities (5 days/week)										
Yes	129 (4.83)	14 (4.14)	Reference	14 (7.21)	Reference	378 (6.60)	51 (7.15)	Reference	48 (8.28)	Reference
No	2708 (95.17)	318 (95.86)	1.33 (0.61, 2.93)	253 (92.79)	1.39 (0.56, 3.42)	5707 (93.40)	751 (92.85)	1.11 (0.77, 1.60)	604 (91.72)	1.02 (0.61, 1.70)
Survey year										
2010	1377 (47.94)	158 (44.75)	Reference	144 (51.47)	Reference	3002 (48.30)	388 (47.54)	Reference	307 (46.17)	Reference
2011	1460 (52.06)	174 (55.25)	1.19 (0.86, 1.66)	123 (48.53)	0.81 (0.50, 1.33)	3083 (51.70)	414 (52.46)	1.00 (0.82, 1.20)	345 (53.83)	1.05 (0.79, 1.39)

ORs adjusted for age, sex, smoking status (never, former, current), family income level, education level, BMI (continuous), physical activity, and survey year.

Table S3. Distribution of serum vitamin D concentrations by year, KNHANES 2008-2014.

**Year**	Overall				Men				Women			
	<10	10-<20	20-<30	≥30	<10	10-<20	20-<30	≥30	<10	10-<20	20-<30	≥30
2008	366 (8.63)	2119 (51.89)	1428 (31.08)	413 (8.40)	71 (5.45)	572 (44.46)	596 (38.45)	202 (11.64)	295 (10.81)	1547 (56.99)	832 (26.02)	211 (6.18)
2009	420 (8.65)	3228 (62.21)	1468 (24.54)	296 (4.59)	82 (5.36)	1050 (59.57)	627 (28.81)	152 (6.26)	338 (10.95)	2178 (64.06)	841 (21.57)	144 (3.43)
2010	384 (9.37)	2625 (60.08)	1126 (25.08)	244 (5.47)	89 (6.58)	822 (54.55)	527 (31.88)	110 (6.99)	295 (11.32)	1803 (63.94)	599 (20.34)	134 (4.40)
2011	298 (6.78)	2933 (66.12)	1164 (24.34)	148 (2.76)	65 (4.71)	941 (61.70)	525 (30.23)	65 (3.36)	233 (8.20)	1992 (69.13)	639 (20.32)	83 (2.35)
2012	353 (8.48)	2786 (67.66)	957 (21.48)	113 (2.39)	83 (5.92)	924 (65.55)	418 (25.65)	48 (2.88)	270 (10.32)	1862 (69.17)	539 (18.47)	65 (2.04)
2013	170 (11.36)	883 (60.21)	359 (23.80)	66 (4.64)	54 (8.84)	361 (59.24)	175 (28.15)	26 (3.77)	116 (13.15)	522 (60.90)	184 (20.69)	40 (5.26)
2014	214 (16.30)	868 (61.76)	272 (18.70)	54 (3.24)	64 (12.11)	339 (61.92)	146 (23.34)	19 (2.63)	150 (19.96)	529 (61.63)	126 (14.64)	35 (3.78)
Total	2205 (9.71)	15442 (61.62)	6774 (24.22)	1334 (4.45)	508 (6.92)	5009 (58.43)	3014 (29.42)	622 (5.23)	1697 (11.70)	10433 (63.91)	3760 (20.49)	712 (3.90)
